# White Men in Quarantine: Disease, Race, Commerce and Mobility in the Pacific, 1872

**DOI:** 10.1080/1031461X.2017.1293704

**Published:** 2017-05-19

**Authors:** Katherine Foxhall

**Affiliations:** University of Leicester

## Abstract

In July 1872, the steamship *Hero* underwent quarantine at Sydney’s North Head after a case of smallpox was diagnosed. This article brings together the histories of quarantine, white subjectivity and Pacific mobility through an analysis of the *Loganiana* newspaper produced by the passengers of the *Hero* during their confinement. The *Loganiana* provides a unique insight into the formation of white identities through discussions of race, commerce, science and inter-colonial politics. The case provides an important perspective on a transformative period in Australia’s border history, and also illuminates the tensions accompanying the transition from an older imperial order to political autonomy in the nineteenth-century Pacific.

## Introduction

**Figure 1.**
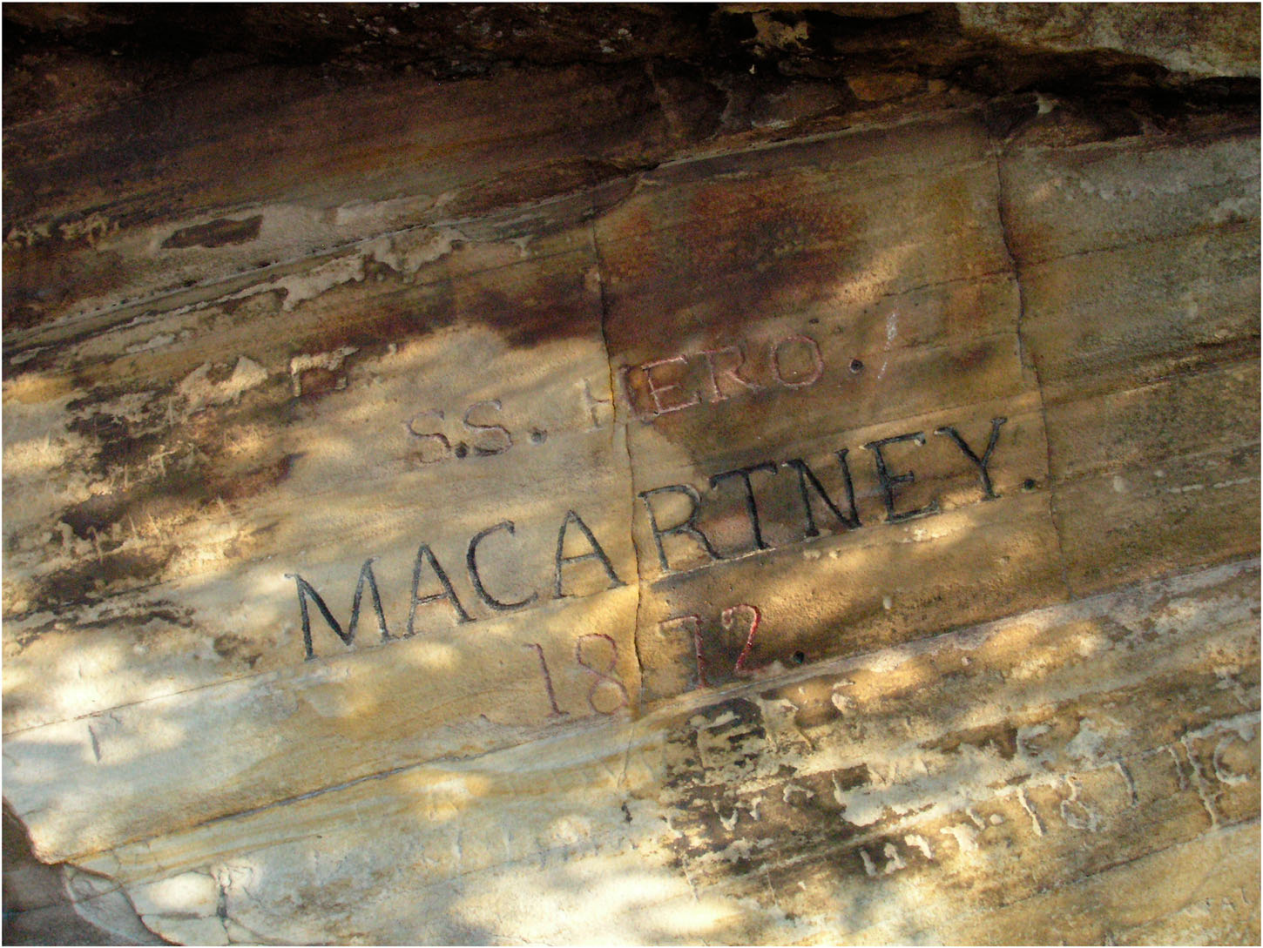
‘Macartney’ carving at North Head quarantine station. Author’s photo.

**Figure 2.**
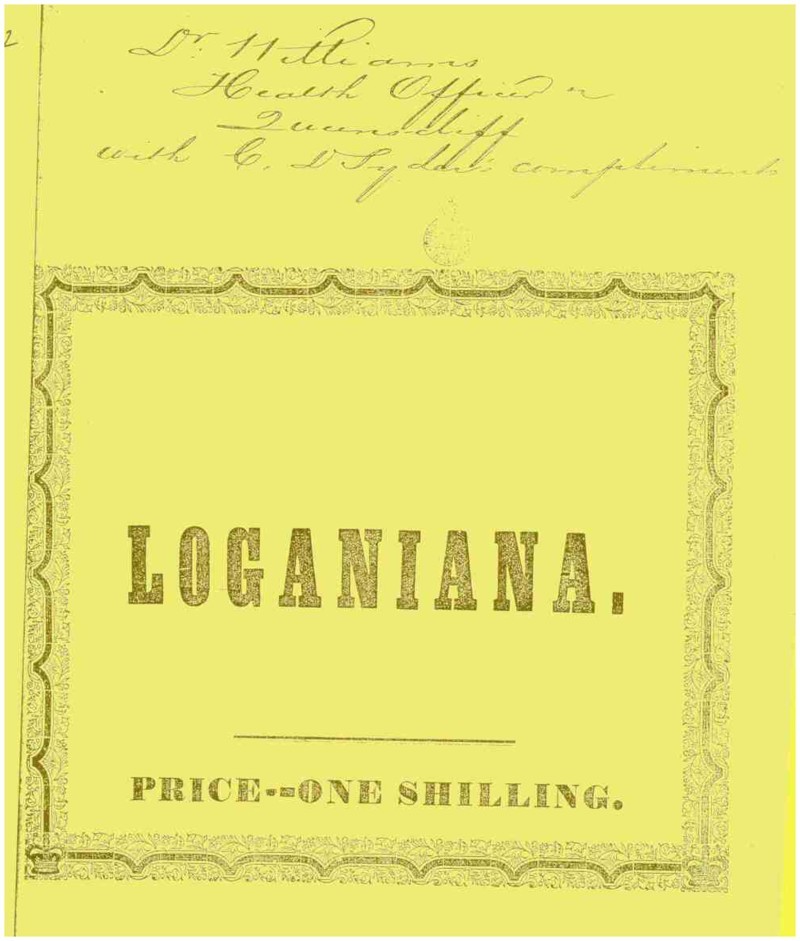
*Loganiana* title-page (1872). Credit: State Library of New South Wales.

In July 1872, the iron passenger steamship *Hero*, owned by the Melbourne mercantile shipping company Bright Bros. and Co., was quarantined at Sydney’s North Head quarantine station after one of the steerage passengers appeared to develop smallpox. A routine fortnightly voyage across the Tasman Sea from Auckland to Melbourne, via Sydney and Newcastle, thus ended with a forty-two-day confinement of all on board at the entrance to Port Jackson. During the quarantine, the *Hero*’s passengers put on shows, wrote poetry, played music and sports, and carved their presence into the North Head rocks (Figure 1). The most striking product of this quarantine, however, was a weekly journal that the passengers compiled in order to relieve the tedium of their ‘imprisonment’. Named *Loganiana* after their captain, Thomas Logan, this was later printed in Sydney. Priced one shilling, and sold by E. Turner, bookseller, on Hunter Street, the souvenir publication had a sulphur-yellow cover reminiscent of quarantine’s yellow flag and the smoke of fumigation (Figure 2).^1^ The *Loganiana* is a rare and fascinating source, and contrasts with many personal accounts of quarantine (such as letters and diaries), by providing a sustained and acerbic commentary on the medical, commercial and cultural politics of quarantine.^2^ Moreover, it illuminates an important moment of transition in the history of quarantine. The *Loganiana* provides a unique opportunity to understand the worldview of a group of elite white men, and how they used fluency in politics, science and culture to articulate the failures of quarantine immediately before the racialisation of border strategies.

Historians have begun to take seriously the racialised and gendered identity of white men, particularly by analysing transnational movements of ideas about race, settlement and government that signalled the emergence of ‘white men’s countries’ or an ‘Anglo-World’ by the twentieth century. This work has shown that a mid-nineteenth-century confidence in a trans-imperial Anglo-Saxon ascendancy gave way to a more defensive rhetoric in response to a changing world order at the turn of the century.^3^ One way that defensiveness manifested was in the development of increasingly rigid immigration laws and racialised border controls, often explicitly aimed against Chinese incomers, in countries such as Australia and the United States. Quarantine had a long history in colonial Australia; it had been used at Sydney from 1814, with Governor Bourke formally declaring the North Head Quarantine Ground in 1837. The 1850s were the busiest years at this station, when at least fifty-nine ships underwent quarantine.^4^ The smallpox epidemic of 1881 marked the shift towards racialised border control that would be enshrined by quarantine’s inclusion in the ‘White Australia’ policy after 1901.^5^

Historians have found Australia’s embrace of quarantine significant, particularly as it diverged markedly from Britain where, following the 1872 Port Health Act, cases of disease that arrived in ports were increasingly dealt with through sanitation and surveillance, rather than quarantine. The British public health ‘zone’ could even stretch to include colonial spaces such as the Suez Canal.^6^ Within continental Europe also, national attitudes towards quarantine were highly sensitive to political and economic interests, imperial rivalries and public opinion. France reduced quarantine in its Mediterranean ports during the 1840s, and by the 1870s, Russia had concluded that it could not enforce quarantine along its vast land borders, particularly with Persia. Other states, notably in the Mediterranean, steadfastly refused to abandon quarantine.^7^ Attempts to reform quarantine and establish multi-lateral cooperation through international sanitary conferences from 1851 resulted in much disagreement and inconsistency, but still laid foundations for international sanitary regulations that aimed to reduce barriers to free movement in the twentieth century.^8^ This contrast with Europe is important, but it is clear that as an aspirationally ‘white’ country, Australia joined New Zealand, Canada and the United States in using fluid racial categories to include, restrict and exclude at the border, a history that also intersects with judgements about class, health, criminality and politics.^9^

The *Loganiana* invites us to bring the histories of quarantine and white subjectivity together, but it also prompts us to shift our focus from Australian quarantine as a national story, to one that is firmly intertwined with Pacific mobility.^10^ As Sydney, Melbourne and Newcastle emerged as the most important Australian seaports by the 1860s, regular steamer services competed for trade along the eastern seaboard, across the Tasman Sea, and beyond into the Pacific. For several years Thomas Logan had captained the *Hero* and its entirely European crew; the whiteness of the ship’s crew is worth noting in an era when South Asian lascars were prominent in global maritime employment.^11^ Logan had recently taken on the route that linked Australia with Auckland, often carrying passengers who had arrived in New Zealand from the Pacific islands and California.

Pacific history has begun to attract considerable attention. While for a long time discussions of cultural encounters in a ‘sea of islands’ had seemingly little connection to macro-economic histories of the northern Pacific rim, and there was little clear sense of how Australia and New Zealand might be included in a ‘southern’ Pacific region, themes of connection are now providing important insights and coherence.^12^ The mid-nineteenth century was certainly a significant time in the Pacific. With new colonies, gold, mining, missionaries and plantation economies, came migration. Steam power transformed possibilities for trans-Pacific travel, enabling people, ideas and goods to cover great distances with a speed, range and lack of restraint inconceivable only a few years earlier. New theories and ideologies spread quickly; racism, for instance, gained real power in the settler Pacific, particularly following the publication of Charles Darwin’s *Origin of Species* in 1859.^13^

The *Loganiana* sheds light on this important moment of transition in the interlinked histories of quarantine, race and the Pacific across a number of scales. The micro-politics of quarantine procedures fed into and upon transpacific webs of communication that fuelled speculation about the nature of the disease. Condemnations of government officials and shipping companies reflected inter-colonial rivalry between New South Wales and Victoria in a post-convict era, but came back to Sydney via a triangular relationship with New Zealand, which also functions in this story as the doorway to disease from the wider Pacific. Shipping routes connecting with America brought the most flamboyant characters to this quarantine, as well as news of world events, and sources with which to discuss issues of race and aboriginal decline.^14^ The *Loganiana* illuminates a period of flux, in which it was by no means clear who the problem ‘other’ was that quarantine was supposed to target. This article argues that in order to understand why and how Australian quarantine came to be aimed at a racial ‘other’ from the 1880s, we also need to bring this history into conversation with the histories of whiteness and the Pacific. We need to understand how, by the 1870s, quarantine became too politically and commercially problematic for white men to use against each other.

## The *Loganiana*

Introducing the *Loganiana*, lawyer and appointed editor Frank Ritchie conceived of the journal as a record of ‘our sayings and doings on board’.^15^ By issue II, he also felt that the newspaper should record the unhappiness of their confinement. Ritchie recognised that governments undoubtedly had a right to issue and enforce stringent regulations to preserve public health, and insisted that the *Loganiana* must not lay the passengers open to charges of unreasonable or exaggerated complaints. Nevertheless, he made it clear that the ship’s company wished to complain about the Medical Officer’s neglect of his duties, ‘and of the inefficient, inconsistent, and arbitrary manner’ in which he had carried out the quarantine regulations. Over the next five weeks, in six issues, the *Loganiana* used satire, verse, waltz, parable, poetry and music to ridicule what they saw as a superstitious quarantine farce. The journal charts the progression of the passengers’ emotions from bemusement and scepticism to disgust and unrestrained anger. The *Loganiana*’s contributors consistently asserted the absence of disease, ridiculed the Sydney authorities responsible for their detainment, discussed politics, and denounced the delays and conditions imposed for their release. They particularly objected to the order that the entire ship’s company submit to vaccination against smallpox before being released. In the final *Loganiana*, Ritchie accepted that some of the ‘real or supposed grievance’ recorded over the previous weeks might appear unreasonable or absurd, but reiterated his belief that the *Loganiana* was an authentic record: ‘a fair expression of the feeling and opinion current in the ship at the time of its publication’.^16^

The *Loganiana*’s tone was driven by a small cast of cabin passengers and senior crew, including Captain Thomas Logan, a ‘Yankee’ with Scottish roots, and other male cabin passengers. Frank Ritchie, a lawyer, became the editor; John Cruickshank, ‘commercial reporter’, was the British government’s vice-consul to the French colony of New Caledonia; Frank Weston, ‘show reporter’, was an American medical showman and minstrel proprietor. The *Hero*’s Liverpudlian purser, R.W. Cogswell, joined as ‘shipping reporter’, and W. Jameson, another male cabin passenger, was ‘law reporter’. These men were fluent in trans-colonial politics, science, literature and law. Non-officer members of the crew, women, and steerage passengers are barely visible in the *Loganiana* except as potential victims of disease and implied audience. The *Loganiana* thus reinscribed conventional elisions of colonial culture as well as the class and gender divisions of ships; in doing so, it lays bare the mental universe of a group of privileged, highly mobile men at a moment of personal vulnerability.

Creating a periodical was a common entertainment for first- and second-class passengers on vessels from Europe to Australia in the late nineteenth century. They commonly recorded entertainments, theatrics and sports, maritime trivia, advertisements, meteorology, natural history and poetry; these were often printed, bound, and sold by subscription as voyage souvenirs. Like other ships’ newspapers, the *Loganiana* fuelled a sense of community and shared experience among a random community in transit, but there is a key difference between the *Loganiana* and other shipboard periodicals; here the journal’s content is driven by the group’s frustrations with an externally imposed immobility, rather than reflecting on their mobility.^17^ That the *Loganiana* certainly went beyond playful satire in its attacks on the authorities responsible for the detention suggests that we should consider this publication alongside the creative collective outputs that have emerged from other confinement contexts. In military hospitals, lunatic asylums, tuberculosis sanatoria and prisoner-of-war camps, communities of insiders have defiantly responded to fear by creating outsiders of those deemed responsible for situations of danger and alienation.^18^

The passengers of the *Hero* exploited the fact that although quarantine is defined by the appearance of isolation, its boundaries could also be extremely porous: words, objects, people and boats continually criss-crossed the harbour-space. Quarantined travellers had long taken advantage of these possibilities for exchange. During the 1830s, emigrant detainees of Sydney’s quarantine station corresponded with local newspapers to challenge negative representations of their status as incomers and immigrants tainted by disease.^19^ During the *Hero*’s quarantine, the passengers similarly responded to newspapers to mitigate the ‘incalculable injury’ being done by the publication of ‘false’ statements. One response was collective: thirty-five of the *Hero*’s passengers signed a letter to the Melbourne *Argus* refuting a report that the *Hero* had been in a filthy condition on a previous trip.^20^ The *Loganiana* drew on and extended these public complaints, and should be understood as situated within this wider history of colonial print culture, a powerful force shaping trans-imperial identities within a global public sphere.^21^

## The identity of disease

The importance of Pacific communication networks in this story is immediately apparent in the flurry of information and speculation about the identity of disease that the *Hero*’s arrival generated. As the *Hero* entered Port Jackson, a *Government Gazette* extraordinary hurriedly ordered the quarantine of all vessels that arrived in Sydney from Victoria and New Zealand as well as those from the Sandwich Islands and California ‘beyond the seas’, for a minimum of seven days.^22^ Colonial newspapers used the new overland telegraph network, as well as information arriving by ship, to immediately cover the story in great detail. On 9 July, a day after the ship’s arrival, the Melbourne *Argus*’s overview of telegraphic despatches named the sick patient as Joseph Sutcliffe, a passenger bound for Melbourne, who before his journey had stayed at an Auckland boarding house where a man had recently died of smallpox. Sutcliffe’s disease was ‘undoubtedly smallpox’, the *Argus* said, introduced at Auckland in June by the *Nebraska*, a US mail steamer that traversed the Pacific from San Francisco via Honolulu*.* The *Argus* had also received ‘an Auckland letter’ explaining how Henry Thompson, a ‘lucky [gold] digger returning to his family in Australia’, had caught smallpox on the *Nebraska* and died in the hospital; a Californian passenger who had left the *Hero* at Auckland had been arrested and quarantined; and ‘Honolulu papers report that the infection prevails there’.^23^

Only the previous week the *Bendigo Advertiser* had implicated the *Hero* in a smallpox outbreak in Bendigo among two families recently arrived from California.^24^ Putting various reports together on 13 July, the *Launceston Examiner* blamed the *Hero* for smallpox outbreaks in New South Wales and Victoria. Noting that Mr Grant, a Main Line Company engineer, had been on the *Nebraska* bound for Tasmania, the *Examiner* hoped that ‘the Government will take some means to keep him out of the colony’, or at least isolate him until the danger had passed.^25^ For readers of Australian newspapers, the apparent ease with which smallpox was hitch-hiking across the Pacific via New Zealand starkly illustrated the weaknesses of an old quarantine system originally set up to deal with sailing ships bringing emigrants from Europe, not the myriad connections of Pacific ports connected by steam. Newcastle, the *Hero*’s original point of arrival in Australia, did not have its own quarantine station. The *Newcastle Chronicle* pointed out that although it was known that there was smallpox in New Zealand and leprosy in Honolulu, the New South Wales government had taken no precautions to prevent the introduction of these ‘dire diseases’. Newcastle, and by extension the whole colony, was ‘entirely unprotected’.^26^

Five days after the *Hero*’s arrival in quarantine, Joseph Sutcliffe died on North Head. Provincial newspapers soon reported that Sutcliffe’s illness had been smallpox ‘of the most virulent type’.^27^ The *Empire* and the *Evening News* both published Frank Ritchie’s furious response to a *Sydney Morning Herald* article entitled ‘Death on board the Hero’ which asserted that Sutcliffe had died on the ship, rather than in the quarantine station. At such a time, ‘it is of the highest public importance’, Ritchie wrote, ‘that unusual precaution should be taken to ensure the accuracy of all such statements’.^28^ At the same time, another story began to circulate: that the man who had stayed in the Auckland boarding house before Sutcliffe had died of ‘Maori pox’. There was some confusion about the significance of this distinction. At the time, some understood Maori pox to be a milder disease than smallpox – perhaps reflecting common assumptions that the excellent New Zealand climate rendered a variety of diseases less severe – and grasped hope from this. The *Illawarra Mercury* hoped that should it be ‘only’ Maori pox, ‘our fair country may still be saved’.^29^ However, other newspapers as far apart as Brisbane and Melbourne quoted Henry Williamson, government vaccinator and surgeon to Berrima gaol, who said that there was no difference in the virulence of ‘aboriginal or European small-pox’, noting that ‘one old man from the Burragorang tribe remembered when several of the different tribes were quite decimated by this disease’.^30^

On the Thursday evening of their third week (18 July), the ‘Heroites’ (as the stranded passengers had begun to call themselves) also discussed the accuracy of Sutcliffe’s smallpox diagnosis. Frank Weston was convinced that the case was not smallpox. Dr Macartney, another cabin passenger, disagreed. He drew on three years’ experience in Dublin hospitals, and supported the government medical officers.^31^ Later that week, however, when one of Macartney’s own children suffered a ‘slight eruption’, his support for the smallpox diagnosis evaporated. Ritchie’s fourth editorial explained that:

The doctor on board seemed to be doubtful as to its nature. The child was taken on shore when the Health Officer arrived, and he was equally doubtful. The next day a third doctor arrived from Sydney, and having examined the child at a respectful distance, pronounced it to be a case of smallpox. Without presuming to question the verdict of a professional doctor, we cannot help regarding it as an extraordinary thing that it should have taken two days and three medical men to arrive at this conclusion. The child is now reported to be perfectly well.^32^

Minnie Macartney’s illness also appeared in the week’s ‘commercial report’ which noted that the *Hero* had received ‘one case of Healthy Rush per Minnie Macartney from Over-eating’. That this had been branded a case of smallpox ‘is the more to be regretted since small-pox is well known to be much more valued by Chemists, Druggists and Medical Men, than is Healthy Rush’.^33^ In ‘Locals’ another contributor suggested to the medical man who had examined the girl on Thursday from a distance of several yards, that ‘in future he need not trouble himself to leave Sydney. With a good Telescope the examinations might be conducted with equal facility from his own residence’.^34^ Frank Ritchie later commented that the *Loganiana* was one child whose death they would welcome, but who ‘has exhibited no intention of dying’.^35^

Reflecting a thriving imperial culture of amateur and professional astronomy fascinated by comets, solar eclipses, sunspots and planetary transits, astronomical bodies were a common feature of the *Loganiana*’s discussions about the existence (or otherwise) of smallpox. The second issue recounted how:

our worthy astronomer Captain Thomas, while examining the sun through a marvellous and intricate instrument, discovered to his horror, a spot on the face of that luminary. He at once pronounced it to be a symptom of smallpox  …  A telegram will be sent to the Health Officer, requesting that he go up in a balloon to examine the patient, and to stay there until wanted on board the *Hero*.^36^

**Figure 3.**
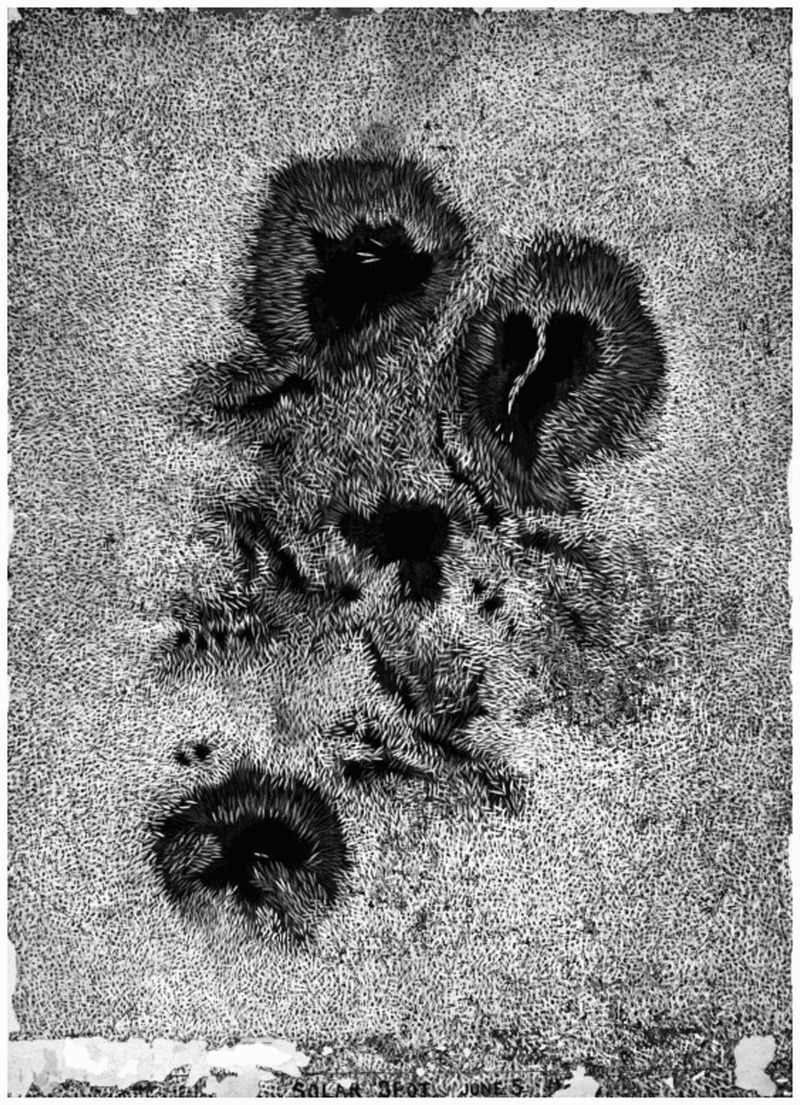
James Nasmyth, solar spot, Science Museum at Wroughton, 1864. Object No. 1956-156. © Science Museum, UK. Image available for use under licence: CC-BY-NC-ND 2.0.

The sun would be the perfect place to banish the credulous Health Officer. Popular contemporary depictions of sunspots, for example by James Nasmyth (Figure 3), bear a striking resemblance to the pitted surface of a smallpox sufferer’s skin, but this was about more than aesthetics. In December 1871, the governments of New South Wales, Victoria and South Australia had helped fund an expedition to Cape York, northern Queensland, to view the solar eclipse. The venture was an abject failure: in the middle of monsoon season, cloud and rain obscured the view. Astronomical humour thus remained a fine topic with which to needle government officials just months later.^37^ Hot air balloons were also a current topic, having been used during the American Civil War and by Parisian citizens during the Prussian siege in 1871.^38^ Ballooning was nevertheless an old science. The *Loganiana*’s suggestion that a balloon might be a suitable mode of transport for the Health Officer presented the rituals of quarantine as archaic and based on unenlightened and unreasonable superstition, at the same time as it confirmed the commentators’ scientific and political fluency. During the fourth week of quarantine the *Hero*’s company put on a Gala and Festival Week. The highlight was a ‘screaming farce’ entitled ‘the Yellow Flag’, with characters such as Vampire Keep-a-way, Lymphatic, Variola Snooks, and Kanker Rash. In another contribution, ‘Dreamer’ described the *Harmony* hulk, moored at the quarantine station next to the *Hero* and used for many years as a hospital ship:

On looking at the craft I saw a pale blue light streaming out of her ports, and the sound of groans was wafted on the night breeze; among other words I heard “small-pox” often repeated. Then different sounds would reach me, such as coughing, sneezing, and blowing of noses. I did not require anyone to tell me that the ghosts of the old quarantinists were going through the old performance of being fumigated. The moon shone out behind a cloud, the pale lights were melted into thin air, and a voice said, “‘Hero,’ ahoy! We know you have no small-pox – these government officials are fools!”^39^

## Race, slavery and decline

**Figure 4.**
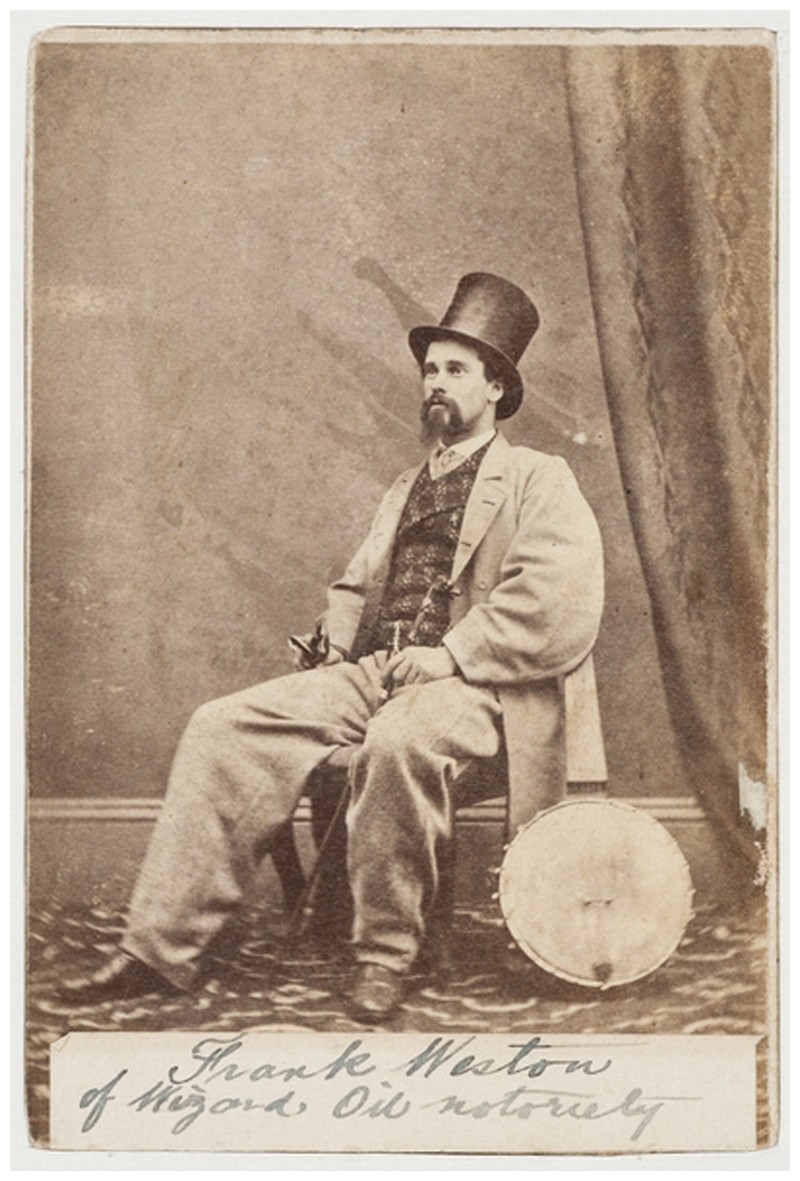
‘Frank Weston of Wizard Oil notoriety’, c. 1865/photographer unknown. Credit: State Library of New South Wales.

The *Loganiana*’s quarantined contributors included two well-known figures. R.G. Bachelder was one of the most successful showmen – many of them American – who toured New South Wales, Victoria, New Zealand, India and South Africa in the 1860s and early 1870s, bringing world events and famous battles such as the Franco-Prussian War and Maori Wars to the viewing public. Since 1867 Bachelder had toured his *American War Panorama* and performance of *Paradise Lost*.^40^ On this journey Bachelder and six members of his troupe had been bringing their ‘Grand Colossean Pantascope Tour through America’ to Australia. The second showman was Frank Weston, the *Loganiana*’s ‘show reporter’ (Figure 4). Weston was the ‘Wizard Oil Prince’, and he capitalised on the Pacific steamship network to bring all the sarcasm and burlesque of the American medicine show phenomenon to the Australasian colonies.^41^ Weston had first peddled his Wizard Oil, Magic Pills and Mexican Mustang Liniment to the Australian public in 1864. By 1870 he was the proprietor of the short-lived Weston’s Opera House in Melbourne and the Weston and Hussey Minstrel Company.^42^ Medicine shows attracted large crowds to provide entertainment and sell patent medicines, but if the character of the racism had changed, blackface performance itself was not new to Australia; the figure of ‘jumping’ Jim Crow had entertained audiences as early as 1839.^43^ Here is a strange conjuncture of quackery and quarantine, two very different medical worlds. Weston may have eased the tedium of the confinement by performing his shows and satire, but even he did not claim that his Wizard Oil – that ‘great American remedy’ for rheumatism, neuralgia and gout – could do anything about smallpox.

The presence of Weston and Bachelder on this voyage reflects an accelerating trans-Pacific exchange of ideas, entertainment and money that fed from the gold rush booms. During the quarantine they provided entertainment, but they also contributed to more serious discussions, and were instrumental in bringing together different ideas about race. On the second Tuesday, the first meeting of the Loganiana Debating Society discussed the subject ‘Slavery, is it right?’ Weston, followed by Dr Macartney and Frank Ritchie, led the affirmative side, maintaining that ‘the idea of all men being born free and equal was a sentimental delusion’. The *Loganiana* reported that Weston evoked ‘the celebrated Mr Darwin’s name, darkly hinting that negroes were little better than monkeys with their tails off’, and that this advantage ‘was due to the simple laws of nature’. Bachelder, Logan and Cruickshank opposed the position. Logan ‘completely rebutted this theory …  on referring to his voluminous memoranda he found that there existed in America a Professor of something or another, who proved  … that in the modifications of species the tail was invariably the last member to drop off’. The debate ended with two questions:

1st Question. – Do you think Slavery right? (Loud Cries of Aye.)

2nd Question. – Do you think Slavery wrong? (Loud Cries of No.)^44^

Weston and the ‘Yankee’ Logan seem to have taken the role of post-Civil War caricatures, representing the southern and northern American states respectively for their audience. The idea of ‘all men born free and equal’, one of the founding principles of the United States Declaration of Independence, and the First Article of the Constitution of Massachusetts (1779), was here being ridiculed, using a distorted invocation of Darwin’s natural laws to support the continuation of slavery. Darwin had recently discussed the absence of tails in *Descent of Man* (1871), in which he argued forcefully that the races of man were all descended from a common progenitor, and in a series of forms that graduated ‘insensibly from some ape-like creature’.^45^ The irony of the *Loganiana*’s distortion of contemporary debates about polygenesis and monogenesis is that Darwin passionately hated slavery; a humanitarian belief in the unity of humanity was the moral force behind his scientific theories.^46^ Again, these debates would have been familiar in Sydney or Melbourne; in 1867 Louis Agassiz’s New York lectures – in which Australia had featured prominently, and in which he argued forcefully for the different origins of the races of mankind – had also been reported in the colonial news.^47^

The discussion subject for the second, and final, meeting of the Debating Society was also about race: ‘Should Aboriginal barbarism or semi-barbarism give way to, and disappear before advanced civilization?’ For many on the *Hero*, that Aborigines were doomed would have seemed obvious, rather than open to question.^48^ Through the nineteenth century, predictions about the extinction of Aboriginal and Maori peoples in Australia and New Zealand had formed part of a much wider debate about colonialism, civilisation, and ‘dying races’. By the 1860s, concern about the condition, and speculation about the fate, of Australian Aborigines fuelled debates about the extent to which differences between human groups were innate or permanent. This was not an abstract issue, but reflected very real problems of colonial dispossession and ongoing frontier violence.^49^ After the ‘conspicuous members’ of the *Hero*’s group handled the subject ‘in their usually vigorous and able manner’, the majority of voices favoured the proposition ‘that Aboriginal barbarism viewed as Josh Billings said of rats, “from any platform you can build is unspeakably cussed”’.^50^

This statement is revealing. Josh Billings was the stage name of Henry Wheeler Shaw, who toured America’s eastern and midwest states, giving talks on ‘Natral History’ in the 1860s and 1870s. In 1868 Shaw had published *Josh Billings on Ice, and Other Things*, a collection of satirical thoughts, sayings and correspondence on diverse subjects including laughing, agriculture, ‘Amerikan Aristokrasy’, and orphan children. In a chapter on rats, Shaw had pronounced: ‘I serpose thare is between 50 and 60 millions of rats in Amerika … and i don’t serpose thare is a single necessary rat in the whole lot  … Rats viewed from enny platform yu kan bild, are unspeakably cussid’.^51^ Here is the precise authority for the Heroites ‘dying race’ proposition. The metaphor of rats would have resonated strongly. In his influential *Handbook of New Zealand Flora* (1864), J.D. Hooker had talked about the ‘replacement’ of native rats, flies, cats and dogs by their European equivalents. This imagery became an important feature of discussions about Maori population decline.^52^ It is striking that this discussion about Aboriginal decline did not refer to well-known antipodean commentators such as Charles Hursthouse, but to an American comic lecturer.

Evolutionary theories and ideas about ‘fatal impact’ gained particular traction in the nineteenth-century Pacific.^53^ In the *Loganiana*, a constellation of racial ideas from an eclectic array of sources came together during the extended sociability occasioned by the *Hero*’s quarantine. In *Drawing the Global Colour Line*, Lake and Reynolds emphasise the circulation of ‘emotions and ideas, peoples and publications, racial knowledge and technologies’ from which the transnational phenomenon of whiteness emerged. While their focus was the influence of key thinkers and political leaders on debates about democracy and self-government, the case of the *Hero* shows how similar processes were at work beyond an intellectual elite, and how racial knowledge in particular could be shaped in highly contingent ways, in this case as an idiosyncratic borrowing of trans-Pacific and imperial preoccupations with race, slavery, and extinction.^54^

## The politics of release

The *Loganiana* provides a striking counter-narrative to the official account of the SS *Hero*’s quarantine in the *Proceedings of the Legislative Assembly* of New South Wales, which stated matter-of-factly that the station had been visited daily (apart from one day), and that the Medical Officer made daily reports to the Health Officer, who then personally reported every second day to the Minister.^55^ By contrast, the *Loganiana* singled out the Health Officer for being conspicuously absent during the early days of the quarantine. Issue III advertised as ‘LOST, by the passengers of the “Hero”, any respect that they may ever have felt for the Health Officer’.^56^ In January 1869, passengers from the *Kaikoura* had complained bitterly to the Legislative Assembly about the want of courtesy, decision and information they received in quarantine. As in the case of the *Hero*, one of their main complaints was that the Health Officer did not visit them.^57^ Through shipping reports, commercial intelligence, editorial comments, amusements and advertisements, the *Loganiana* also attacked the New South Wales Legislative Council, and the Sydney-based Australasian Steam Navigation Company that competed directly with the *Hero*’s Victorian owners for passengers and trade. Issue II asked whether the Executive Council consisted ‘of M.Ds or A.S.Ses’ for imposing just a six-day quarantine on rival ship *St Nicholas* which had arrived from California, when the regulations stipulated a minimum of seven days for a ship with no sickness on board.^58^

The fourth issue of the *Loganiana* reported the Health Officer’s announcement that once everyone on board had been vaccinated against smallpox, the ship would be released. Though Ritchie believed that the stipulation was preposterous, given the length of time that had elapsed since Sutcliffe’s infection (smallpox incubated for up to seventeen days), he nevertheless regretted that some of the *Hero*’s passengers had refused to submit. He pointed out that as soon as they had done what was required, the ship would have to be relieved as promised. Vaccination was blackmail, but by refusing, some of the passengers gave the Health Officer an opportunity, ‘of which there is little doubt he will avail himself’, to blame them for their own detention.^59^ In any case, each day during the next ‘wasted’ week the Health Officer arrived without the necessary lymph to vaccinate the passengers. The *Loganiana* decried ‘the dilatory and shilly-shallying behaviour of this gentleman who professes to be exceedingly anxious to obtain our release’.^60^

The passengers of the *Hero* who refused the vaccine took a stand against the conditions of their detention, but they were also in an unusual legal position. Tasmania and Victoria had implemented compulsory vaccination legislation during the 1850s, and New Zealand had made vaccination compulsory for infants from 1863. Although New South Wales had provided voluntary vaccination from 1847 through a Vaccine Institute situated next to the Hyde Park Barracks, it had never followed suit on compulsion, despite considerable pressure. Indeed, even as the *Hero* lay at North Head, the Legislative Council admitted that while they ‘were adopting stringent measures to prevent the introduction of smallpox into the colony’, the government did not actually have the power to compel vaccination.^61^ This thorny question of consent would re-emerge in later quarantines.

From the start of the quarantine, the *Loganiana*’s contributors directed some of their ire at ‘Piddlington’, the ‘commander-in-chief of the ASN corps’. This was Richard Piddington, a director of the Australasian Steam Navigation Company, colonial treasurer, member of the Legislative Assembly, and signatory of the government’s quarantine proclamations of 8 July. He was responsible for apprehending the passengers who had left the *Hero* at Newcastle and escorting them into quarantine. Such an overt conflict of medical politics and commercial interest infuriated the *Hero*’s passengers, who believed their suppression was solely down to the ‘cool intrepidity’ and crafty forethought of this man. In issue III of the *Loganiana*, ‘Impertinence’ enquired whether there was any member of the government of New South Wales who was also a director of the ASN Company, and in whose interest it would be to keep the *Hero* in quarantine as long as possible. ‘Certainly not, Sir!’, came the sarcastic response. An advertisement proclaimed ‘FOUND, that it makes all the difference to a ship in quarantine, whether she belongs to the A.S.N. Company or not’.^62^

The fifth issue’s ‘Shipping Report’ described the Heroites watching the arrival of the ASN steamer *Alexandra* from the infected port of Auckland, with the *Nebraska* mails and passengers from California and the Sandwich Isles on board. In contrast to the *Hero*, which by this time had been detained for over a month, the seemingly favoured vessel of the local New South Wales company steamed straight past the quarantine station and in to Sydney Wharf, despite its origin in Auckland, and its risky contacts, not least with the *Nebraska* which had been implicated in Joseph Sutcliffe’s original case of smallpox. ‘Did you ever hear of such corruption in a government?’ one of the *Hero*’s ‘prisoners’ asked in a letter reported by the Auckland newspaper, the *Daily Southern Cross.*^63^

The following week, ‘Answers to Correspondents’ explained that since the detention of the *Hero*, the ASN had ‘reduced’ their fares on the route between Sydney and Auckland from £10 to £14. Laden with sarcasm, the Commercial Report warned that when such ‘Incivility and Grinning Mockery’ were pushed upon a market, ‘their forced sale is always speedily followed by transactions in Contempt and Disgust’ and ‘the illicit manufacture of Indignation, Alarm, and even Rage and Despair’. John Cruickshank, the commercial reporter, called upon ‘our’ authorities at the quarantine station to prohibit and prevent ‘any transactions whatever with ticket-of-leave men and old convicts from Sydney’.^64^ Although desperate to escape their ‘pestilential settlement’ the Heroites claimed moral high ground on the headland as they ridiculed the ‘nightly scenes of atrocity, pillage, garotting, and sometimes murder’ that occurred in ‘the old penal settlement’ of Sydney.

Finally, after six weeks, the *Hero* was released from quarantine. A month later, on the occasion of Captain Logan’s first return to New Zealand after the quarantine, the Mayor of Auckland held a banquet in his honour, attended by the principal merchants and citizens of the town. In his toast, the Mayor described the quarantine as a ‘persecution’ animated by the jealousy of the Australian Steam Navigation Company, and the conduct of the New South Wales government as ‘arbitrary and cruel’. During the dinner, Logan’s Auckland friends presented him with ‘an elegant silver salver and claret jug’, to commemorate the quarantine.^65^ Some months later, these objects appeared on public display, not in Auckland or Melbourne, but in Sydney, in Friederich Allerding’s shop on Hunter Street.^66^ Allerding was a chronometer maker and photographer, and held great scientific authority in Sydney. In 1874, he would contribute to the report on the Transit of Venus.^67^ By publicly placing these objects dedicated to Logan at the heart of Sydney’s booming commercial, maritime and scientific community, the citizens of New Zealand pointedly informed the New South Wales government – and its public – of their distaste. Their snub in Allerding’s shop used the cultural cachet of science and commercial networks to make a political point about the offence quarantine caused to sophisticated men who expected to freely traverse the Tasman Sea.

## Conclusion

Quarantine emerged from the saga of the *Hero* as an arbitrary procedure riddled with absurdities, irregularities and inter-colonial commercial jealousies. The debates about disease on the *Hero* brought together three issues: whether or not indigenous peoples and Europeans had suffered the same or different kinds of pox since the arrival of the First Fleet in 1788; the necessity of vaccine legislation; and the failures of quarantine to deal with trans-Pacific connections. Perhaps more uncomfortably for the Legislative Council, it became clear that quarantining white men was becoming a commercial and political, as well as medical, problem. The eclectic range of subjects covered by the *Loganiana* echoes Tony Ballantyne’s discussions of ‘cultural traffic’, and the exchange of knowledge about science, race, commerce, politics and print in the late nineteenth century.^68^ Yet these discussions – and particularly the jokes – about commerce, politics and science, reinforced particular kinds of social relationships and knowledge in which only a few of the passengers could fully participate.

The final issue of the *Loganiana* recorded a visit to the quarantine station by Mr Syder, the representative of the *Hero*’s Melbourne-based owners, the Bright Brothers. The correspondent remarked that it was pleasant ‘after so long in a barbarous country to behold once more the face of one who hails from a white country’. The *Sydney Illustrated News* was unimpressed with the implications of this slur. Commenting that ‘such an expression may be all very witty in itself’, it suggested that the quarantine flag had ‘cast its jaundiced reflection over the better nature of at least one member of the party’.^69^

The references to Sydney as a barbarous ‘old penal settlement’ and Victoria as ‘a white country’ emphasise that the *Hero*’s quarantine occurred at a transitional moment, sandwiched between the penal history of an ‘old’ colonial Australia and the emergence of a ‘new’ racialised national border in an increasingly connected Pacific region. It also provides an opportunity, as Penelope Edmonds has recently urged, to pay attention to the ‘spatiality’ of whiteness, and to the language that people used in racialising places as well as bodies.^70^ It is significant that while the discourse surrounding the *Hero* did conflate race and disease, it was through comments about ‘Maori’ smallpox, rather than making links to Chinese migration. Even as late as 1876, there would be a surprising lack of xenophobic sentiment when the *Brisbane*, with the majority of its crew made up of Chinese and lascar sailors, became implicated in a smallpox scare.^71^ In 1878–9 the Australian Steam Navigation Company became the target of protests and strikes, after the company employed cheap Chinese labour.^72^ This episode came to play a key role in Australia’s intertwined history of labour and race relations, making the absence of Chinese discussion in the *Loganiana* even more striking, particularly given the centrality of the ASN to the passengers’ quarantine critique. It shows just how quickly the emergence of a nationalist racist quarantine would occur in the early 1880s; this was not an inevitable or gradual growth, but reactive to rapidly changing circumstances. In the early 1870s these circumstances had looked very different.

Despite the Heroites’ protests, white men (and women) on suspect ships continued to undergo quarantine at North Head throughout the 1870s, until the focus shifted to Chinese arrivals from 1881. But the case shows how those people whose interests the quarantine affected – both within and far beyond the station – mobilised scientific, commercial and political arguments to stress the failures of quarantine in an age of expanding transnational mobility, and a formative period of regional Pacific identities.^73^ The *Loganiana*’s critique of quarantine was shaped by the myriad transpacific connections that people, objects and illnesses had made, and it also provides a unique insight into the ideas that informed white masculinity – from discussions of slavery and indigeneity to trade and astronomy. The racial discussions and jokes recorded in the *Loganiana* certainly reflect a shared sense of confidence and destiny – but this is also a story of vulnerability and uncertainty, in which rival white identities turned on each other, challenging each other’s motives, boundaries, and authority. The *Loganiana* is thus revealing of the tensions that accompanied the transition from an older imperial order to political autonomy in the nineteenth-century Pacific.

## Footnotes

1 At least two copies of the *Loganiana* survive: in the Mitchell Library, State Library of New South Wales (the copy used here, ref. ML Mss F910.42), and National Library of New Zealand.

2 Krista Maglen, ‘Quarantined, Exploring Personal Accounts of Incarceration in Australian and Pacific Quarantine Stations in the Nineteenth Century’, *Journal of the Royal Australian Historical Society* 91, no. 1 (2005): 1–14.

3 Key studies include Marilyn Lake, ‘On Being a White Man, Australia, c.1900’, in *Cultural History in Australia*, eds Hsu-Ming Teo and Richard White (Sydney: University of New South Wales Press, 2003), 98–112; Marilyn Lake and Henry Reynolds, *Drawing the Global Colour Line: White Men’s Countries and the International Challenge of Racial Equality* (Cambridge: Cambridge University Press, 2008); James Belich, *Replenishing the Earth: The Settler Revolution and the Rise of the Anglo-World, 1783–1939* (Oxford: Oxford University Press, 2009).

4 Jean D. Foley, *In Quarantine: A History of Sydney’s Quarantine Station, 1824–1984* (Sydney: Kangaroo Press, 1995), 155–7.

5 Alison Bashford, ‘Quarantine and the Imagining of the Australian Nation’, *Health* 2, no. 4 (1998): 387–402, 388, 397–8.

6 Krista Maglen, ‘A World Apart: Geography, Australian Quarantine and the Mother Country’, *Journal of the History of Medicine and the Allied Sciences* 60, no. 2 (2005): 196–217; Krista Maglen, *The English System: Quarantine, Immigration and the Making of a Port Sanitary Zone* (Manchester: Manchester University Press, 2014), 82–3.

7 Peter Baldwin, *Contagion and the State* (Cambridge: Cambridge University Press, 2009), 213–14.

8 A good overview of these debates is Mark Harrison, ‘Disease, Diplomacy and International Commerce: The Origins of International Sanitary Regulation in the Nineteenth Century’, *Journal of Global History* 1 (2006): 197–217.

9 Alison Bashford, ‘Immigration Restriction: Rethinking Period and Place from Settler Colonies to Postcolonial Nations’, *Journal of Global History* 9, no. 1 (2014): 26–48.

10 Frances Steel, ‘Re-Routing Empire? Steam-Age Circulations and the Making of an Anglo Pacific, c. 1850–1890’, *Australian Historical Studies* 46, no. 3 (2015): 356–73.

11 Ravi Ahuja, ‘The Age of the “Lascar”: South Asian Seafarers in the Times of Imperial Steam Shipping’, in *Routledge Handbook of the South Asian Diaspora*, eds Joya Chatterji and David Washbrook (London: Routledge, 2014), 110–22.

12 This is exemplified in David Armitage and Alison Bashford, eds, *Pacific Histories: Ocean, Land, People* (Basingstoke: Palgrave Macmillan, 2014); Frances Steel, *Oceania under Steam: Sea Transport and the Cultures of Colonialism, c.1870–1914* (Manchester: Manchester University Press, 2011); Donald Denoon, Philippa Mein Smith with Marivic Wyndham, *A History of Australia, New Zealand and the Pacific* (Oxford: Blackwell, 2000).

13 See chapters by James Belich and Adam McKeown in Armitage and Bashford*.*

14 I use ‘transpacific’ as proposed by Frances Steel, denoting networks and exchanges ‘formative of settler colonial imaginaries of the Pacific and the Australian colonies’ place within them’: Steel, ‘Re-Routing Empire?’, 358.

15 Frank Ritchie, Introduction to *Loganiana*, I, 12 July 1872, 1.

16 Frank Ritchie, Editorial, *Loganiana*, VI, 10 August 1872, 1.

17 Fariha Shaikh, ‘*The Alfred* and *The Open Sea*: Periodical Culture and Nineteenth-Century Settler Emigration at Sea’, *English Studies in Africa* 57, no. 1 (2014): 21–32; Ronald Wenzlhuemer and Michael Offermann, ‘Ship Newspapers and Passenger Life Aboard Transoceanic Steamships in the Late Nineteenth Century’, *Transcultural Studies* 3, no. 1 (2012): 77–121, 104–6.

18 Annabelle Winograd, ‘Ten Minutes of Anthrax! Some Notes on French Combatant Trench Scripts of the First World War’, *Theater* 31, no. 1 (2001): 51–69; Christine Winter, ‘The Long Arm of the Third Reich: Internment of New Guinea Germans in Tatura’, *The Journal of Pacific History* 38, no. 1 (2003): 85–108.

19 Katherine Foxhall, *Health, Medicine and the Sea: Australian Voyages, c. 1815–1860* (Manchester: Manchester University Press, 2012), 202–5.

20 ‘The SS Hero’, *Argus*, 23 July 1872, 1.

21 Christopher Holdridge, ‘Circulating the African Journal: The Colonial Press and Trans-Imperial Britishness in the Mid-Nineteenth-Century Cape’, *South African Historical Journal* 62, no. 3 (2010): 487–513.

22 *New South Wales Government Gazette*, 8 July 1872, 1.

23 ‘Telegraphic Despatches’, *The Argus*, 9 July 1872, 5.

24 ‘Small-Pox in Long Gully’, *Bendigo Advertiser*, 6 July 1872, 2.

25 ‘Smallpox in N.S.Wales and Victoria’, *Launceston Examiner*, 13 July 1872, 7.

26 ‘The Newcastle Chronicle’, *The Newcastle Chronicle*, 9 July 1872, 2.

27 E.g. ‘New South Wales’, *Adelaide Observer*, 20 July 1872, 3.

28 ‘SS Hero in Quarantine. To the Editor of the Empire’, *Empire*, 19 July 1872, 2.

29 ‘The Illawarra Mercury’, *Illawarra Mercury*, 16 July 1872, 2.

30 ‘Maori Small-pox’, *Sydney Morning Herald*, 16 July 1872, 3.

31 ‘The Wizard Oil Prince’, *Loganiana*, III, 20 July 1872, 2.

32 Editorial, *Loganiana*, IV, 27 July 1872, 1.

33 ‘Commercial Report’, *Loganiana*, IV, 27 July 1872, 1

34 ‘Locals’, *Loganiana*, IV, 27 July 1872, 1.

35 Frank Ritchie, Editorial, *Loganiana*, IV, 27 July 1872, 1.

36 ‘Locals’, *Loganiana*, II, 15 July 1872, 2.

37 Raymond Haynes, *Explorers of the Southern Sky: A History of Australian Astronomy* (Cambridge: Cambridge University Press, 1996), 55–7; Alex Soojung-Kim Pang, ‘The Social Event of the Season: Solar Eclipse Expeditions and Victorian Culture’, *Isis* (1993): 252–77.

38 C.H. Gibbs-Smith, ‘Notes on the History of Ballooning’, *Journal of Navigation* 10, no. 1 (1957): 63–7.

39 ‘The “Harmony” Hulk’, *Loganiana*, IV, 27 July 1872, 2.

40 Melissa Bellanta, ‘Leary Kin: Australian Larrikins and the Blackface Minstrel Dandy’, *Journal of Social History* 42, no. 3 (2009): 677–95, 682; Mimi Colligan, *Canvas Documentaries: Panoramic Entertainments in Nineteenth-Century Australia and New Zealand* (Melbourne: Melbourne University Press, 2002), 70, 73–82.

41 James Harvey Young, *The Toadstool Millionaires: A Social History of Patent Medicines in America before Federal Regulation* (Princeton: Princeton University Press, 1961).

42 Richard Waterhouse, *From Minstrel Show to Vaudeville: The Australian Popular Stage, 1788–1914* (Sydney: New South Wales University Press, 1990), 43.

43 ‘Royal Victoria Theatre’, *Sydney Monitor and Commercial Advertiser*, 1 November 1839, 3; Clare Anderson, *Subaltern Lives: Biographies of Colonialism in the Indian Ocean World, 1790–1920* (Cambridge: Cambridge University Press, 2012), 61–5.

44 ‘Debating Society’, *Loganiana*, II, 15 July 1872, 2.

45 Charles Darwin, *The Descent of Man, and Selection in Relation to Sex*, vol. 1 (London: John Murray, 1871), 156, 235, www.gutenberg.org/files/34967/34967-h/34967-h.htm#Page_9 (accessed 7 May 2015).

46 Adrian Desmond and James Moore, *Darwin’s Sacred Cause: Race, Slavery and the Quest for Human Origins* (London: Allen Lane, 2009).

47 ‘Professor Agassiz on Men and Monkeys’, *Empire*, 30 May 1867, 3.

48 Warwick Anderson, *The Cultivation of Whiteness: Science, Health and Racial Destiny in Australia* (New York: Basic Books, 2003), 195–8; Philippa Levine, ‘Anthropology, Colonialism, and Eugenics’, in *The Oxford Handbook of the History of Eugenics*, eds Philippa Levine and Alison Bashford (Oxford: Oxford University Press, 2010), 43–61.

49 Toeolesulusulu Damon Salesa, ‘“The Power of the Physician”: Doctors and the “Dying Maori” in Early Colonial New Zealand’, *Health and History* 3 (2001): 13–40; Leigh Boucher and Lynette Russell, ‘“Soliciting Sixpences from Township to Township”: Moral Dilemmas in Mid-Nineteenth-Century Melbourne’, *Postcolonial Studies* 15, no. 2 (2012): 149–65; Benjamin Madley, ‘From Terror to Genocide: Britain’s Tasmanian Penal Colony and Australia’s History Wars’, *Journal of British Studies* 47, no. 1 (2008): 77–106.

50 ‘Debating Society’, *Loganiana*, IV, 27 July 1872, 1.

51 Henry Wheeler Shaw, *Josh Billings on Ice and Other Things* (New York: Carleton, 1868), 199–202.

52 Ross Galbreath, ‘Images of Colonisation: Native Rats and Dying Pillows’, *The Turnbull Library Record* xxv (1993), 33–42, cited in Salesa, 37–8.

53 James Belich, ‘Race’, in Armitage and Bashford, 263–81, 268–9.

54 Lake and Reynolds, 5–10; Belich, *Replenishing the Earth*, 324.

55 New South Wales. *Votes and Proceedings of the Legislative Assembly during the Session 1872*, vol. 1 (Sydney, 1872), 260.

56 ‘Advertisements’, *Loganiana*, III, 20 July 1872, 2.

57 ‘Hardships of Quarantine’, *Empire*, 9 January 1869, 3. My thanks to Peter Hobbins for telling me about *Kaikoura*.

58 ‘Local’, *Loganiana*, II, 15 July 1872, 2.

59 ‘Advertisements’, *Loganiana*, IV, 27 July 1872, 2.

60 Editorial, *Loganiana*, V, 3 August 1872, 1.

61 ‘The Parliament. Summary of Proceedings’, *The Sydney Mail and New South Wales Advertiser*, 20 July 1872, 86.

62 ‘Answers to Correspondents’, *Loganiana*, III, 20 July 1872, 2; Alison Bashford, *Imperial Hygiene: A Critical History of Colonialism, Nationalism and Public Health* (Basingstoke: Palgrave Macmillan, 2004), 51–7.

63 ‘The “Hero” in Quarantine’, *Daily Southern Cross*, 10 August 1872, 2.

64 ‘Commercial Report’, *Loganiana*, V, 3 August 1872, 1.

65 ‘Banquet to Captain’, *Daily Southern Cross*, 19 September 1872, 3.

66 ‘Inter-Provincial Shipping’, *West Coast Times*, 8 January 1873, 2.

67 Henry Chamberlain Russell, *Observations of the Transit of Venus, 9 December 1874* (Sydney: Charles Potter, 1892), 39.

68 Tony Ballantyne, ‘Race and the Webs of Empire: Aryanism from India to the Pacific’, *Journal of Colonialism and Colonial History* 2, no. 3 (2001), https://muse.jhu.edu/journals/journal_of_colonialism_and_colonial_history/v002/2.3ballantyne.html (accessed 13 May 2015).

69 *Illustrated Sydney News*, 31 August 1872, 2.

70 Penelope Edmonds, ‘“I Followed England Round the World”: The Rise of Trans-Imperial Anglo-Saxon Exceptionalism, and the Spatial Narratives of Nineteenth-Century British Settler Colonies of the Pacific Rim’, in *Re-Orienting Whiteness*, eds Leigh Boucher, Jane Carey and Katherine Ellinghaus (London: Palgrave, 2009), 99–115, 101–3.

71 Peter Hobbins, ‘Union Jack or Yellow Jack? Smallpox, Sailors, Sovereignty and *Salus Populi*’ (forthcoming 2017 research article).

72 Denoon *et al*., 175; Ann Curthoys, ‘Conflict and Consensus’, *Labour History* 35 (1978): 48–65.

73 For quarantines in the 1870s see Foley, 157–8.

